# Successful management of TSH-secreting adenoma misdiagnosed as hypothyroidism in 65-year-old male: A case report

**DOI:** 10.1016/j.ijscr.2025.111153

**Published:** 2025-03-14

**Authors:** Ahmed Sheikh Sobeh, Kenana tawashi, Ayham Qatza, Shahama Al-shami, Wardan A. Tamer

**Affiliations:** a Faculty of Medicine, Hama University, Hama, Syria; b Oncologist resident, Al Bairwni Hospital, Al Mouasat Hospital, Damascus, Syria; c Endocrinologist, Hama, Syria

**Keywords:** TSHoma, Pituitary tumors, Hyperthyroidism, Case report

## Abstract

**Introduction:**

Thyrotropin-secreting adenoma (TSHoma) is a rare functional pituitary tumor, accounting for less than 3 % of all pituitary tumors, characterized by central hyperthyroidism and inappropriate TSH secretion.

**Presentation of case:**

We present a case of a 65-year-old man with a history of unresponsive hypothyroidism, diagnosed with a pituitary tumor through MRI and CT scans. Following partial tumor resection, histopathological examination confirmed a benign TSHoma. The patient was discharged in stable condition after three days, receiving long-term octreotide injections and propranolol without complications.

**Discussion:**

TSHomas, resulting from abnormal growth of TSH-producing pituitary cells, pose diagnostic challenges and are often misdiagnosed. *Trans*-Sphenoidal surgery is the standard treatment, and long-acting somatostatin analogs like octreotide can effectively restore normal thyroid function in over 90 % of patients.

**Conclusion:**

This report emphasizes the need to consider TSHoma as a potential cause that may be initially overlooked, highlighting the diagnostic and management challenges of this rare condition.

## Highlights

• Thyrotropin-secreting adenoma (TSHoma) is a rare functional pituitary tumor, accounting for less than 3 % of all pituitary tumors.
• TSHomas, resulting from abnormal growth of TSH-producing pituitary cells, pose diagnostic challenges and are often misdiagnosed.
• This report emphasizes the need to consider TSHoma as a potential cause of hyperthyroidism.

## Abbreviations

TSHoma: Thyrotropin (TSH)-secreting adenoma
FT4: Free thyroxine 4
FT3: Free triiodothyronine
MRI: Magnetic resonance imaging
CT: Computed tomography
SITSH: Syndrome of inappropriate secretion of thyrotropin
TSS: Trans-sphenoidal surgery

## 1 Introduction

Thyrotropin (TSH)-secreting adenoma (TSHoma) represents a rare type of functional pituitary tumor characterized by excessive TSH production, resulting in elevated serum thyroid hormone levels [1]. It accounts for less than 3 % of all pituitary tumors [2]. Central hyperthyroidism and pituitary lesions are the main manifestations of TSHoma, showing typical hyperthyroid symptoms and inappropriate TSH secretion. In addition, pituitary macroadenomas may cause visual field defects and vision loss [1]. Diagnosis of TSHoma involves clinical manifestations and signs, endocrine hormone levels, functional tests, and imaging findings. Elevated free thyroxine 4 (FT4) and free triiodothyronine (FT3) serum levels with non-suppressed TSH indicate potential TSHoma after exclusion of laboratory testing interference [3]. The European Thyroid Association guidelines advocate trans-sphenoidal or subfrontal adenectomy as the initial approach for restoring thyroid function [4,5]. However, for patients not cured by surgical management, medical therapies such as somatostatin analogs and radiotherapy may be considered [6]. Herein, we discuss a patient's case previously diagnosed with hypothyroidism and treated unsuccessfully with thyroxine. Subsequent evaluation revealed TSHoma, and it was treated with the surgery. This work has been reported in line with the SCARE Criteria [7].

## 2 Case presentation

**Fig. 1:**
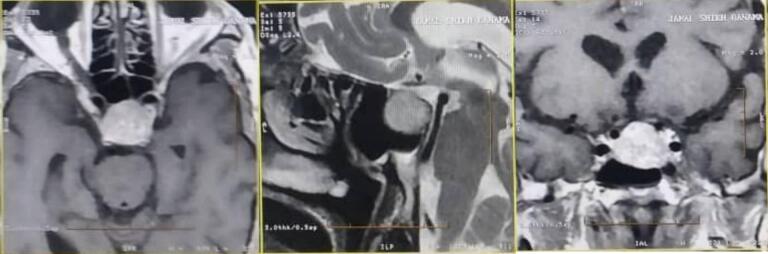
Brain MRI (horizontal, sagittal, and coronal planes) showing pituitary’ tumor.

A 65-year-old man was referred to our endocrine clinic, complaining of general fatigue, anorexia, weight loss, severe vomiting without nausea, and palpitations from a year ago. The patient's medical history showed that he was diagnosed with hypothyroidism a year ago and was treated with thyroxin for ten months without any response (the diagnosis was made by a local doctor depending on elevated TSH levels without T4 and T3 levels equalization). The patient referred to gastroenterology and hematology specialists to exclude other possible differential diagnoses, and they declared that there was not any gastroenterology or hematology problem. The psychosocial history showed that he was smoking (without determining the amount and timing). The surgical, familial, and drug (except for thyroxin) histories were unremarkable. The patient's vital signs were normal except for an increased heart rate (118 b/m). His physical examination revealed only generalized pallor. The laboratory investigations were as follows [Table 1]. The brain magnetic resonance imaging (MRI) showed the pituitary gland's tumor [Fig. 1]. Therefore, a high suspicion of TSHoma was there. We referred the patient to a specialist in neurosurgery to decide on the best treatment choice. We put him on octreotide (0.1 mg under cutaneous, three times a day). The neurosurgeon declared that the tumor should be excised so, computed tomography (CT) imaging was done; its findings were similar to that of brain MRI. The patient was also prepared for surgery with hydrocortisone and achieved euthyroidism (FT4: 1.58 ng/dl, FT3: 3.96 pg/dl). The cardiovascular and pulmonary consultations showed no contraindications to the surgery. The tumor was removed partially (due to infiltrating into the cavernous sinus) through a microscopic trans-sphenoidal approach. The histopathological examination of the tumor showed a benign TSHoma. The laboratory tests after 24 h of the surgery revealed euthyroidism, and the patient was discharged from the hospital in a healthy situation after 3 days on long-term octreotide injection and propranolol (10 mg, twice a day). The patient's follow-up, depending on laboratory tests, showed a recurrence of hyperthyroidism [Table 2] as the patient did not take the octreotide because of the hard economic situation. Once the patient took the octreotide, euthyroidism was achieved. After three months of surgery, the patient had weight gain (13 kg increased) without any complications such as diabetic insipidus or pituitary gland insufficiency.

**Table 1:** laboratory investigations' values.

WBCs	Neutrophils	Lymphocytes	Monocytes	RBCs
3.51 × 10^3^/mm^3^↓	1.83 × 10^3^/mm^3^	1.40 × 10^3^/ mm^3^	0.28 × 10^3^/ mm^3^	2.64 × 10^6^/mm^3^↓
HB	HCT	MCV	PLT	Phosphorus
8.3 g/dl↓	22 %↓	83.33 fl	90 × 10^3^/mm^3^↓	3.97 mg/d
Calcium	Sodium	Potassium	Glucose Fasting	IGF-1
9.96 mg/dl	139.6 mmol/l	4.19 mmol/l	91 mg/dl	16 ng/ml↓
TSH	T4 (total)	FT4	FT3	Cortisol (8 am)
26.6 ulU/ml ↑	21.44 μg/dl↑	3.10 ng/dl↑	5.21 pg/dl↑	8.56 μg/dl
Prolactin	Widal	Wright's		
16.6 ng/ml	Negative	Negative

**Table 2:** the patient laboratory values in the first and twice weeks and after a month.

One week after the surgery	Free T4 = 2.11 ng/dl ↑	TSH = 11.2 ulU/ml ↑
Two weeks after the surgery	Free T4 = 2.29 ng/dl ↑	
Month after the surgery	Free T4 = 2.11 ng/dl↑	TSH = 17.7 ↑

## 3 Discussion

Hyperthyroidism is a pathological state resulting from an abnormal increase in the synthesis and secretion of thyroid hormones by the thyroid gland [8]. Autoimmune thyroid disorders, toxic thyroid nodules, and goiters are the usual causes of hyperthyroidism. Nonetheless, TSHoma, caused by abnormal clonal growth of the TSH-producing pituitary cells, can be the cause in rare cases [6,9]. TSHoma represents 0.5–3 % of pituitary tumors and is usually misdiagnosed [8,9]. The prevalence of TSHoma is found in up to 0.94 % of all pituitary adenomas [6]. The connection between the pituitary and extreme TSH production causing secondary hyperthyroidism was proposed in 1960 [9]. TSHoma was confirmed in case reports in 1980 [9]. TSHoma typically presents with mild thyrotoxicosis symptoms and neurological complications from pituitary mass compression. TSHoma is identified by somatostatin receptors, particularly SSTR2 and SSTR5 [8]. Between 1990 and 2009, the rate of TSHoma diagnoses increased, with the incidence rising from 0.05 to 0.26 cases per million individuals per year [6]. The diagnosis of TSHomas as a contributing factor to hyperthyroidism presents significant challenges. Indeed, it is typically indicated by the observation of the syndrome of inappropriate secretion of thyrotropin (SITSH), which manifests as persistently unsuppressed elevated TSH levels in conjunction with elevated thyroid hormone levels [6]. Elevated TSH levels in the presence of hyperthyroidism should prompt consideration of a concomitant TSHoma; specific biochemical markers, including a high alpha subunit/TSH ratio, elevated TSH alpha-subunit, and a reduced response of TSH to TRH stimulation, exhibit sensitivities of 83 %, 75 %, and 71 %, respectively [6]. In a review encompassing 535 cases of TSHoma, the median TSH level at diagnosis was recorded at 5.16 mU/l [6]. Diagnostic imaging modalities employed in evaluating TSHomas include pituitary MRI, radiolabelled somatostatin receptor scintigraphy, and positron emission tomography scans [6]. The majority of TSHoma cases are characterized by the presence of pituitary macroadenomas in imaging studies. Two systematic reviews examining patients with TSHoma have indicated that up to 79 % of these individuals exhibit macroadenomas [6]. In our condition, we report the case of a 65-year-old man with a benign TSH-secreting tumor-type macroadenoma and the challenges we faced to reach the correct diagnosis, starting with the misdiagnosis with hypothyroidism by a local doctor and treatment with thyroxin for ten months without any response depending on elevated TSH levels. Then, after stopping thyroxine and repeating the tests, and with the help of MRI, it was determined that there was a pituitary tumor. The tumor was excised by a neurosurgeon and sent for pathology, which confirmed a TSH-secreting pituitary tumor of the macroadenoma type. The diagnosis and treatment of TSHomas are detailed in the European Guidelines published in 2013 and updated in 2019 [9]. *trans*-Sphenoidal surgery (TSS) is considered the standard approach for both diagnosing and treating TSHomas. The primary method for treating TSHomas is through trans-sphenoidal pituitary resection, and using radiotherapy alongside surgery can provide additional benefits. For effective medical management, long-acting somatostatin analogs like octreotide are recommended, as they can restore normal thyroid function in over 90 % of patients [9]. The administration of long-acting somatostatin analogs, such as octreotide, for a minimum of two months aids in the differential diagnosis of central hyperthyroidism and the treatment of TSHoma. Additionally, a case report has documented successful TSHoma treatment using pasireotide as an alternative somatostatin analog [8]. In our case, we opted for surgical excision of the tumor, for which we administered preoperative doses of corticosteroids. Postoperatively, the patient was managed with octreotide injections in conjunction with propranolol. Follow-up assessments indicated a marked improvement in the patient's overall condition, with no complications reported.

## 4 Conclusion

This report presents a patient exhibiting distinct biochemical and clinical signs of hyperthyroidism. It underscores the importance of considering TSHoma as a potential underlying cause, which may initially be overlooked in such cases. Further investigations confirmed the presence of TSHoma. This case serves as a reminder for clinicians to carefully interpret thyroid function tests when diagnosing hyperthyroidism. Additionally, it highlights the challenges associated with the diagnosis and management of this rare condition.

## Registration of Research Studies

1. Name of the registry:

2. Unique identifying number or registration ID:

3. Hyperlink to your specific registration (must be publicly accessible and will be checked):

## Methods

This work has been reported in line with the CARE criteria [9].

## Consent for publication

Written informed consent was obtained from the patient for publication of this case report and any accompanying images. A copy of the written consent is available for review by the Editor-in-Chief of this journal on request.

## Ethics approval and consent to participate

Ethics clearance was not necessary since the University waives ethics approval for publication of case reports involving no patients' images, and the case report is not containing any personal information. The ethical approval is obligatory for research that involves human or animal experiments.

## Guarantor

Kenana tawashi.

## Funding

The author(s) received no financial support for the research, authorship, and/or publication of this article.

## CRediT authorship contribution statement

**Ahmed Sheikh Sobeh:** Project administration, Conceptualization, Data curation, Writing – original draft, Writing – review & editing. **Kenana tawashi:** Data curation, Writing – original draft, Writing – review & editing. **Ayham Qatza:** Data curation, Writing – original draft, Writing – review & editing. **Shahama Al-shami:** Supervision, Investigation, Writing – review & editing. **Wardan A. Tamer:** Supervision, Investigation, Writing – review & editing.

## Competing interest

The author(s) declared no potential conflicts of interest with respect to the research, authorship, and/or publication of this article.

## Data Availability

We would like to share our raw data. All data generated or analyzed during this study are included in the published article and its supplementary information files.
